# Patterns of Snow Leopard Site Use in an Increasingly Human-Dominated Landscape

**DOI:** 10.1371/journal.pone.0155309

**Published:** 2016-05-12

**Authors:** Justine Shanti Alexander, Arjun M Gopalaswamy, Kun Shi, Joelene Hughes, Philip Riordan

**Affiliations:** 1 The Wildlife Institute, School of Nature Conservation, Beijing Forestry University, Beijing, China; 2 Department of Zoology, University of Oxford, Oxford, United Kingdom; 3 Wildlife Without Borders UK, Oxfordshire, United Kingdom; 4 Statistics and Mathematics Unit, Indian Statistical Institute - Bangalore Centre, Bengaluru, India; 5 Eco-Bridge Continental, Beijing, China; University of Tasmania, AUSTRALIA

## Abstract

Human population growth and concomitant increases in demand for natural resources pose threats to many wildlife populations. The landscapes used by the endangered snow leopard (*Panthera uncia*) and their prey is increasingly subject to major changes in land use. We aimed to assess the influence of 1) key human activities, as indicated by the presence of mining and livestock herding, and 2) the presence of a key prey species, the blue sheep (*Pseudois nayaur*), on probability of snow leopard site use across the landscape. In Gansu Province, China, we conducted sign surveys in 49 grid cells, each of 16 km^2^ in size, within a larger area of 3392 km^2^. We analysed the data using likelihood-based habitat occupancy models that explicitly account for imperfect detection and spatial auto-correlation between survey transect segments. The model-averaged estimate of snow leopard occupancy was high [0.75 (SE 0.10)], but only marginally higher than the naïve estimate (0.67). Snow leopard segment-level probability of detection, given occupancy on a 500 m spatial replicate, was also high [0.68 (SE 0.08)]. Prey presence was the main determinant of snow leopard site use, while human disturbances, in the form of mining and herding, had low predictive power. These findings suggest that snow leopards continue to use areas very close to such disturbances, as long as there is sufficient prey. Improved knowledge about the effect of human activity on large carnivores, which require large areas and intact prey populations, is urgently needed for conservation planning at the local and global levels. We highlight a number of methodological considerations that should guide the design of such research.

## Introduction

Growing pressures of human populations and concomitant rises in demand for natural resources are rapidly fragmenting remaining habitats and putting some wildlife populations at risk [1–5]. Large carnivores, which require large areas and intact prey populations, are especially under threat [2,6]. Some carnivores, such as pumas (*Puma concolor*) and leopards (*Panthera pardus*), appear able to adapt to human-modified environments [4,7], in particular by shifting their diets from wild prey to domestic dogs and livestock [4]. These shifts are taking place in a context of direct conflicts over space or livelihoods, which have in the past led to the elimination of carnivores from human-dominated landscapes [8]. Our understanding of, and responses to these human-wildlife interactions, will sway whether a species survives [1].

The endangered snow leopard (*Panthera uncia*), subsisting in a seemingly isolated and remote landscapes, is potentially at risk to human disturbances [9]. The snow leopard, amongst the least studied of the big cats, has a vast global range, spread throughout the mountains of central and western Asia. The terrain used by snow leopards is often regarded as high, remote, isolated, and largely undisturbed by humans [10]. Yet this environment is increasingly accessible to economic development and subject to major changes in land use through livestock herding, human settlements, road building, mining and hydrological developments [11,12]. China, where economic growth has reached unprecedented levels in the last 3 decades, holds an estimated 60% of suitable snow leopard habitat [9] across 7 different provinces [13]. There is little understanding, however, of how snow leopards are using human-altered landscapes [10].

Previous studies on the interaction between humans and snow leopards have largely focused on community attitudes and potential conflicts around livestock grazing and depredation [14–17]. In contrast, the response of snow leopards to development activities such as the building and use of mines, dams and roads has received relatively little attention. Such knowledge would inform measures to minimize the impact of habitat loss and improve the connectivity and viability of remaining snow leopard populations [18].

In this study, we applied occupancy modelling to assess landscape-scale probability of site use by snow leopards in a selected area of the Qilian Mountain range, Gansu Province, China. This work aimed to extend and scale up our previous occupancy work on snow leopards in a smaller part of the Qilianshan mountains [19]. Occupancy modelling incorporates the probability of detection into the estimation of occupancy or habitat use, overcoming the potential bias related to false absences [20]. To capture the effects of local disturbances at a fine spatial scale within an expected snow leopard home range, we estimated probability of snow leopard use of a given site, instead of the “true occupancy” of a given area [21,22].

Early work compared the frequency of signs across different habitats and regions [23,24]. More recent surveys have drawn on habitat models of various types, including regressions [25], resource-selection functions [26], analysis of utilization-availability data with VHF collar data [27] and habitat suitability ranking [28]. However, these approaches do not assess snow leopard distributions or site use in the face of imperfect detections, which may lead to underestimation of the true spatial distribution [20,29].

In this study, we investigated key factors influencing the probability of snow leopard site use across the northern part of the Qilianshan National Nature Reserve (QNNR). Specifically we sought to: 1) assess how snow leopard site use varies across the landscape in relation to human activities, as indicated by the presence of mining and livestock grazing, and 2) examine the role of a key prey species, the blue sheep (*Pseudois nayaur*), on probability of site use. We assumed that prey occurrence (as measured through recording blue sheep signs) would largely describe habitat quality, and so we did not explicitly include this important determinant of snow leopard site use.

We hypothesized that snow leopard populations largely confine themselves to undisturbed expanses of the nature reserve, with less frequent or no use of areas affected by human disturbances such as mining and livestock rearing. Livestock rearing (of yak and small stock such as sheep and goats) is the primary local livelihood and land use activity within the region and associated presence of herding dogs and humans [30]. We also hypothesized that the availability of suitable prey increased the probability of snow leopard site use.

## Materials and Methods

### Ethics Statement

China’s State Forestry Administration reviewed all sampling procedures and approved permits for the work conducted in QNNR. Non-invasive methods were applied and approval from an Institutional Animal Care and Use Committee or equivalent animal ethics committee was therefore not required.

### Study Area

The study site is located in the northern part of the snow leopard range in China, at the edge of the Qilianshan mountains of Sunan Yugur Autonomous County, Zhangye Prefecture, Gansu Province, China. The northern slopes of the mountains, located in Gansu Province, fall within the transitional zone between the Tibetan Plateau (4000 to 5000 m in altitude) and the Inner Mongolian Plateau (1000 to 2000 m in altitude) [31]. The QNNR area, which covers an area of 26,530 km^2^, supports carnivores including the snow leopard, brown bear (*Ursus arctos*), Eurasian lynx (*Lynx lynx*), grey wolf (*Canis lupus*), red fox (*Vulpes vulpes*) and dhole (*Cuon alpinus*) [32], while blue sheep and white-lipped deer (*Przewalskium albirostris*) are the main wild ungulates within the area [30].

This research was carried out in the northern region of QNNR, known as Qifeng, at altitudes ranging from 1800–4800 m. Human activities are widespread within QNNR. These include herding livestock, principally yak, sheep and goat [30] and mining of coal, jade, iron ore and gypsum. Wire fencing demarcates pastures throughout the reserve. Several roads, some sealed, others graveled, also pass through the reserve. Two sealed roads are used on a daily basis all year round by four-wheeled vehicles crossing the reserve and accessing development projects. The graveled roads are used less frequently to access more remote areas and mines. The graveled roads are relatively insignificant in the larger landscape, with infrequent and highly seasonal use. A number of villages are located just outside the northern boundary of QNNR, two small settlements are located within the reserve and herder huts are scattered throughout the landscape.

### Sampling Design

We conducted field surveys during winter, from January to March 2014, since this season offers easier access along frozen rivers. Late winter and early spring also coincide with the snow leopard mating season, which could possibly lead to increased activity of males across wider areas than in other times of the year.

**Fig 1:**
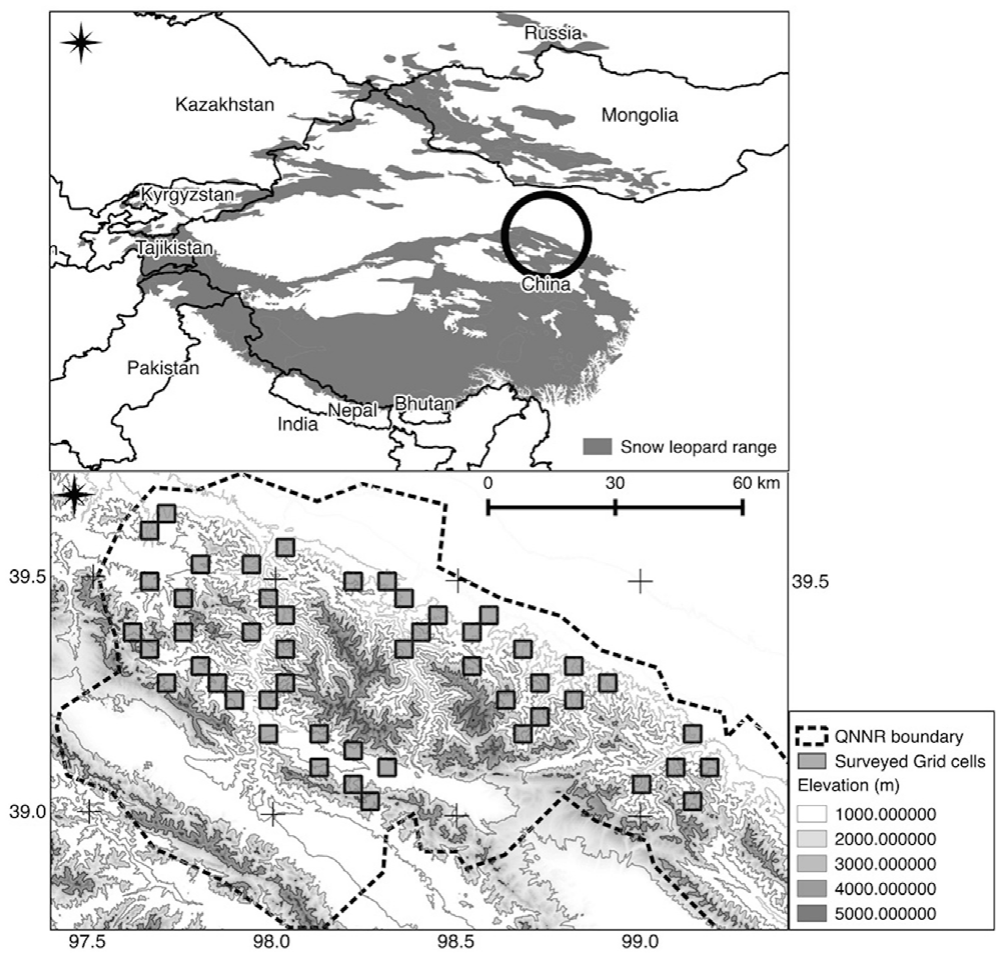
Study Area. Qilianshan National Nature Reserve, Gansu Province, China showing the Nature Reserve boundary and the 49 grid cells surveyed from January to March 2014. The grey polygon in the top map represents the snow leopard range within China estimated by the International Union for Conservation of Nature [35]

We used QGIS [33] to overlay a matrix of 16 km^2^ grid cells onto the centre of Qifeng (Fig 1). We chose to assess the probability of snow leopard site use of different areas of Qifeng and not “true occupancy”. We therefore selected a grid cell size of 16 km^2^ at the lower end of published home range estimates, 11–142 km^2^ [27,34]. Based on our previous work [19] this grid cell size appears to provide adequate spatial coverage despite the logistical challenges posed by the mountainous terrain.

We discarded incomplete grid cells on the periphery and retained 448 (7168 km^2^) grid cells that were entirely contained within the reserve boundary. Of the 448 grid cells, we identified 212 that is 47%, and covering 3392 km^2^, that were within 6 km from a sealed or passable gravelled road and could therefore be surveyed within a single day. Of these 212 grid cells, we randomly selected and surveyed 49 grid cells (784 km^2^). If a grid cell was deemed inaccessible to humans on arrival, for example due to natural barriers, we selected the closest accessible grid cell. The inferences of the analysis are restricted to the 47% of grid cells that are nearest to roads.

Within each of the 49 grid cells, we conducted sign surveys along a distance of 3–7 km within a day. A continuous transect route was planned beforehand in order to minimize spatial autocorrelation and ensure adequate spatial coverage of the grid cells. We selected routes that snow leopards were most likely to use, including ridgelines, valleys and natural pathways [34], in order to maximise snow leopard detection rates.

Each kilometre of the transect route was divided into 10 x 100 m contiguous transect segments and signs of presence of mammalian carnivores were sought in every segment. For each 100 m transect segment, we noted and identified carnivore signs (tracks, scrapes), prey species and livestock (pellets, dung and tracks) and classified them according to species. Additional signs of the same species in the same 100 m segment were not recorded separately.

We only recorded signs considered to be recent (< 1 month old) with sharply defined edges and shape. We attributed signs to different species on the basis of size, shape and context-specific information. Correct identification of species is critical to ensure the robustness of occupancy modelling. Snow leopard tracks were distinguished from those of other carnivores on the basis of criteria recommended by the Snow Leopard Handbook, including track size, shape and other features [36]. We differentiated the tracks of domestic goats and sheep signs from those of wild ungulates using contextual evidence, such as the presence of known pastures, trails/travel routes and the associated tracks of humans, given that small stock are always accompanied by herders in this setting. Our field guide, a local herder, provided in-depth knowledge of herding practices in the study site, and used the size and shape of pellets as further confirmation. We only recorded and retained signs that were clearly visible and unambiguously identified as belonging to a specific species. Weather conditions during the fieldwork were stable (with only 3–4 days of light snow over the 90 day period).

Signs attributed to snow leopard detected in each 100 m segment were converted into the standard detection histories (‘1’ for detection or ‘0’ for non-detection) required for occupancy analyses [37,38]. Sign detection data were then aggregated for each 500 m length, so that each 500 m acted as a spatial replicate and thus reduce zero inflation in the dataset.

### Sampling of Covariates

We restricted the number of covariates to 3 in order to focus the assessment on potential critical human threats and take into account our small sample size (49 grid cells). The selection of covariates was based on previous research, field observations and discussions with key informants from the Nature Reserve staff. Data collected in the field served to create indices characterizing the presence of prey (blue sheep) and of livestock (yak, goat and sheep). Our indices of prey and livestock presence were the proportion of 100 m transect segments in each grid cell in which we detected relevant signs following the methods of Karanth et al. (2011) [39].

We generated an additional index to characterize other predominant human disturbances in this region: distance to mines. We identified all mines within the study area and in the surrounding zones up to 10 km from the QNNR border. Mines were defined as surface excavation sites of an area greater than 4.0 ha made in the earth for the purpose of extracting coal, iron ore, gypsum or precious stones. Only active mines, recognized by local people and the presence of heavy machinery or people, were included. We were able to visually identify most (20/28) of the mines in the field. A number of small-scale mines were also observed but not included within this analysis due to their small size (< 0.5 ha). We calculated the closest distance (in km) from grid cell centroid to active mines.

All covariates were standardized prior to analysis [40] and we explored correlations amongst our covariates using the Pearson correlation test, and considered variables with |r| > 0.7 as highly correlated [41].

### Occupancy Modelling

To select the appropriate occupancy model and reduce the number of candidate models, we applied a three-stage model-building approach within the Program PRESENCE version 8.8 [42]. Akaike Information Criterion adjusted for small samples (AICc) was used to rank models. Given the size of our grid cells and the possibility that snow leopards may have home ranges that overlap multiple sites, we interpreted the parameter occupancy (*ψ*) as the probability of snow leopard site use.

In the first step, we assessed whether the Hines et al. (2010) occupancy model (henceforth referred to as the correlated detection model), which explicitly addresses spatial auto-correlation of sign detections made along spatial replicates, was appropriate for our data set. We used the correlated detection model [38] in which we assume that the local occupancy probability of the first segment (because we have no information on the occupancy state of the previous segment) is a random outcome of the stationary distribution of the Markovian correlated detection model. We compared the MacKenzie’s single season occupancy model [20], abundance-induced heterogeneity model [43] and the correlated detection model [38] to choose the appropriate model type. We included the Royle and Nichols (2003) model as we suspected that the effect of abundance-induced heterogeneity would be large, given our small grid cell size (and consequently low site-specific abundance). As expected, the correlated detection model worked best for our data, based on AIC weight, and was used for further analysis [39].

In the second step, we held occupancy constant under a global model that included all three *a priori* selected covariates (*Mine+ Blue sheep+ Livestock*). Detection probability (*p*_*t*_) was then fitted as a function of these covariates following the method described by Karanth et al. (2011). We expected that all three covariates might influence site use, and also detection probability through their influence on level of site use. We did not identify any other factor that might heavily influence detection in this setting, and this step of the analysis did not support the use of any additional covariates, given our sample size. We used the best detection model in the third and final modelling step [39] and tested the effect of different combinations of covariates on occupancy. Originally our intent was to also investigate the effect of co-variates on the segment-level occupancy parameters (*θ*,*θ'*,*θ*_0_). However the models did not support additional covariates and therefore we were not able to include any covariates for segment-level occupancy parameters in any of the analyses.

For all further analysis we only considered models with >0.01 AIC weight [44]. We assessed the relative importance of each parameter by summing its AIC weights across all models in which it occurred, summed AIC weight [45]. Estimates of coefficients for covariates (*β*) were used to determine effect size and direction of influence.

First we computed model-specific occupancy and detection estimates by model averaging. We calculated the mean occupancy estimate for each model following the equation $\left(\hat{\bar{\psi \;}}=\frac{{\sum^{\text{​}}}_{i=1}^{49}{\hat{\psi }}_{i}\;}{49}\right)$, where ${\hat{\psi }}_{i}$ was defined as the estimated snow leopard occupancy rate for a cell *i* [39]. The standard error estimate of the mean occupancy rate ($\hat{\bar{\psi \;}}$) was calculated using parametric bootstrapping [39] (see S1 File). The same procedure was used to calculate the mean detection estimate (${\hat{\bar{\text{p}}}}_{t}\left(i\right)$) and its corresponding standard error for each model. We then calculated cell-specific estimates of occupancy and final averaged occupancy, detection and segment-level occupancy parameters across all grid cells based on model AIC weights and calculated unconditional standard error estimates [45].

Finally, to test the strength of our inferences based on the estimates, we simulated data using program GENPRES [46], using parameter estimates that we obtained during estimation, for sample sizes comparable to our study (see S2 File).

## Results

### Distribution of Detections

Fig 1 shows the distribution of the sampled grid cells in relation to topography. Sampled grid cells covered a wide range of terrain conditions throughout the study area with the exception of the two high altitude (> 5,000 m) and inaccessible to humans central and eastern zones. The mean elevation of the sampled grid cells ranged from 2199 m to 4246 m (mean 3348 m; SD 486). We surveyed a total of 244 km of 500 m spatial replicates in 49 grid cells covering an area of 784 km^2^. Each grid cell contained a mean of 9.92 (SD 1.82) 500 m spatial replicates (range: 6–14).

Blue sheep was the most frequently detected species, with signs detected in 37 grid cells and in 32% of 500 m transect segments. Snow leopard signs were detected in 33 grid cells (67% naïve occupancy, that is, the proportion of sites in which snow leopards were detected) and in 24% of 500 m transect segments. Livestock activity was less widespread, with livestock signs found in 23 of the grid cells, but in only 15% of the 500 m transect segments. Among livestock species, yak was detected in more grid cells than small stock (16 and 11 grid cells, respectively). The mean distance of the centre of grid cells to the nearest mine was 10 km (SD 6) and the minimum distance was 760 m. The elevation of mines included in the analysis ranged from 1919–3401 m (mean = 1885 m, SD 271).

### Occupancy Model Selection

Covariates were not strongly correlated (all Pearson correlation coefficients < 0.30). We noted however that livestock and blue sheep presence were negatively correlated at a 10% α-significance level (Pearson correlation, r = —0.26).

In the first step, the correlated detection model fitted the data better than the Royle and Nichols (2003) model and MacKenzie’s single season occupancy model, based on AICc values, with an AIC weight close to 1 (Table 1). In the second step, snow leopard segment-level detection probability (P_*t*_) was best explained by the model that included mining as a covariate (AICwt = 0.33). This suggests that detection was highest in areas further away from mines (*β* value = 1.26, SE 0.66). Therefore, all further occupancy model analyses used distance to mines as a covariate to assess detection.

**Table 1:** Summary of model selection results; role of covariates in determining snow leopard detection and site-use probability on 500 m long spatial replicates used in the field survey conducted in Qilianshan National Nature Reserve, 2014. Number of sites = 49.

	Model *	AICc	dAICc	AICwt	Model Likelihood	K	LL
	Step 1						
1	Ψ(.)θ(.) θ’(.) p_t_(.) θ_0_(.)	431.31	0.00	1.00	1.00	5	421.31
2	λ(.).r(.) *	451.86	20.55	0.00	0.00	2	447.86
3	Ψ(.)p(.)	477.78	46.47	0.00	0.00	2	473.78
	Step 2						
1	Ψ(global),θ’(.),θ(.),θ_0_(.),p_t_(M)	421.04	0.00	0.33	1.00	9	398.42
2	Ψ(global),θ’(.),θ(.),θ_0_(.),p_t_(.)	421.78	0.74	0.23	0.69	8	402.18
3	Ψ(global),θ’(.),θ(.),θ_0_(.),p_t_(BS)	422.16	1.12	0.19	0.57	9	399.54
4	Ψ(global),θ’(.),θ(.),θ_0_(.),p_t_(M+BS)	423.15	2.11	0.12	0.35	10	397.36
5	Ψ(global),θ’(.),θ(.),θ_0_(.),p_t_(L)	424.77	3.73	0.05	0.15	9	402.15
6	Ψ(global),θ’(.),θ(.),θ_0_(.),p_t_(BS+L)	425.00	3.96	0.05	0.14	10	399.21
7	Ψ(global),θ’(.),θ(.),θ_0_(.),p_t_(M+BS+L)	425.74	4.70	0.03	0.10	11	396.60
8	Ψ(global),θ’(.),θ(.),θ_0_(.),p_t_(M+L)	433.02	11.98	0.00	0.00	10	407.23
	Step 3						
1	Ψ(BS),θ’(.),θ(.),θ_0_(.),p_t_(M)	417.73	0.00	0.46	1.00	7	401.00
2	Ψ(BS+L),θ’(.),θ(.),θ_0_(.),p_t_(M)	418.71	0.98	0.28	0.61	8	399.11
3	Ψ(M+BS),θ’(.),θ(.),θ_0_(.),p_t_(M)	419.87	2.14	0.16	0.34	8	400.27
4	Ψ(global),θ’(.),θ(.),θ_0_(.),p_t_(M)	421.04	3.31	0.09	0.19	9	398.42
5	Ψ(L),θ’(.),θ(.),θ_0_(.),p_t_(M)	430.28	12.55	0.00	0.00	7	413.55
6	Ψ(.),θ’(.),θ(.),θ_0_(.),p_t_(M)	430.80	13.07	0.00	0.00	6	416.80
7	Ψ(L+M),θ’(.),θ(.),θ_0_(.),p_t_(M)	433.05	15.32	0.00	0.00	8	413.45
8	Ψ(M),θ’(.),θ(.),θ_0_(.),p_t_(M)	433.41	15.68	0.00	0.00	7	416.68

* Covariates considered Mine (M), Blue Sheep (BS) and Livestock (L).

Ψ: the probability of snow leopard site use

θ’: Probability a snow leopard use of a transect segment conditional on snow leopards did use the previous segment

θ: Probability a snow leopard use of a transect segment conditional on snow leopards did not use the previous segment

θ_0_: Probability a snow leopard use of the first transect segment conditional on the segment before the first segment is occupied

p_t_: Probability of detecting a snow leopard in a transect segment conditional on snow leopards used the transect segment

AICc: Akaike’s Information Criterion adjusted for small sample size

dAICc: Change in AICc

AICwt: AIC weight

K: Number of parameters

LL: 2log-likelihood

Note that the Ψ, θ’, θ and θ_0_ parameters are sometimes held constant under the ‘global’ model (*Mine+Blue sheep+ Livestock*)

### Influence of Covariates on Probability of Site Use

Blue sheep presence was found to have the greatest influence on snow leopard site use (summed AICwt = 1.00; Table 2), present in all four top candidate models. Based on the *β* estimates, snow leopards appear to visit more frequently the sites with greater blue sheep presence (Table 2). Based on the top model, estimated $\hat{\psi }$ increased from 0.20, 0.88 to 1.00 as the prey index increased from 0.2, 0.4 to 0.6 respectively (i.e., when prey signs were observed in 20%, 40% and 60% of 1 km transect segments, respectively). The second ranked covariate was the presence of livestock grazing activity (summed AICwt = 0.37), which had a negative influence on probability of snow leopard site use. The third ranked covariate, distance to mines (summed AICwt = 0.25), had a slight positive influence on snow leopard site use. The predictive power of livestock grazing activity and distance to mines should be interpreted with caution, however, given the large SE.

**Table 2:** Estimates of *β* coefficient values for different covariates hypothesized to influence snow leopard site use in Qilianshan National Nature Reserve, 2014.

	Model *	$\hat{\beta }\left[\hat{S}E\right]$	$\hat{\beta }\left[\hat{S}E\right]$	$\hat{\beta }\left[\hat{S}E\right]$	$\hat{\beta }\left[\hat{S}E\right]$
		Intercept	Mine	Blue sheep	Livestock
1	ψ(BS),θ’(.),θ(.),θ_0_(.),p_t_(M)	3.04 (1.74)	-	6.66 (3.45)	-
2	ψ(BS+L),θ’(.),θ(.),θ_0_(.),p_t_(M)	3.88 (2.46)	-	8.21 (4.80)	-1.51 (1.33)
3	ψ(BS+M),θ’(.),θ(.),θ_0_(.),p_t_(M)	3.38 (1.86)	1.03 (1.22)	7.27 (3.64)	-
4	ψ(global),θ’(.),θ(.),θ_0_(.),p_t_(M)	4.21 (2.70)	1.04 (1.29)	8.84 (5.21)	-1.59 (1.39)
	Relative parameter importance (Summed AICwt)	1.00	0.25	1.00	0.37

* Covariates considered Mine (M), Blue Sheep (BS) and Livestock (L).

ψ: the probability of snow leopard site use

θ’: Probability a snow leopard use of a transect segment conditional on snow leopards did use the previous segment

θ: Probability a snow leopard use of a transect segment conditional on snow leopards did not use the previous segment

θ_0_: Probability a snow leopard use of the first transect segment conditional on the segment before the first segment is occupied

p_t_: Probability of detecting a snow leopard in a transect segment conditional on snow leopards used the transect segment

AICwt: AIC weight

Note that the ψ parameter is sometimes held constant under the ‘global’ model (*Mine+Blue sheep+ Livestock*)

**Fig 2:**
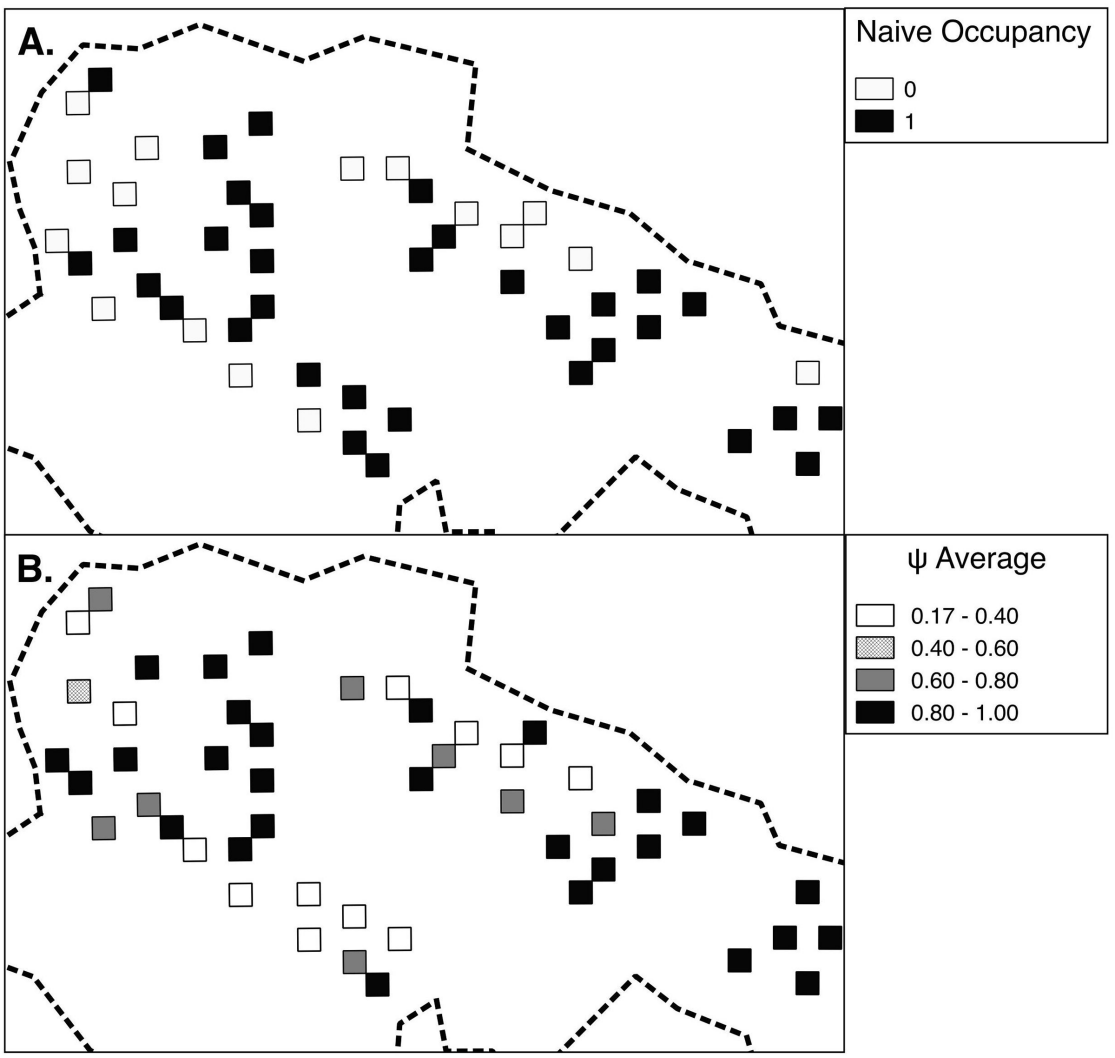
Map of snow leopard site use probability as measured by sign surveys conducted in Qilianshan National Nature Reserve, Gansu Province, China, January to March 2014. A. Naïve estimate of site use (*ψ*) from presence vs absence approach and B. Average estimated probabilities of site use ($\hat{\psi }$) per grid cell.

As expected detection was more likely in transect segments that followed those where snow leopards had been detected (${\hat{\theta }}^{\prime }$ = 0.87, $\hat{SE}$ 0.07), than in segments that followed those with no detection ($\hat{\theta }$ = 0.19, $\hat{SE}$ 0.04). Our estimate that the segment prior to the first in a series was occupied was ${\hat{\theta }}_{0}$ = 0.18 ($\hat{SE}$ 0.16). Our model averaged estimate of occupancy across all grid cells was high at 0.75 (SE 0.10), 11% greater than the naïve estimate (S3 File). The model-averaged snow leopard transect-level detection probability was also high at 0.68 (SE 0.08) per 500 m searched. Variations in grid cell $\hat{\psi }$ showed a range of high and low habitat-use probabilities (Fig 2).

Our simulation results showed that the quality of our occupancy estimate derived from 49 sampled sites needs improvement (RMSE = 0.07). This could be achieved by increasing the number of sites and replicates (See S2 File).

## Discussion

It is recognised that threats to snow leopards are often context-specific. There is therefore an urgent need for rigorous and practical assessment methods to appraise threats at the local level [47]. In 2013, we conducted a camera trap study in a smaller part of QNNR (480 km^2^) to explore possible determinants of snow leopard site use and highlighted the need for further assessments at a larger scale [47,48]. Here, we present such an appraisal of the Northern section of QNNR covering several thousand square kilometres. Our results suggest that snow leopards are present over wide areas of the Qilian mountains, while there is evidence of a range of human activities across the landscape. We also underline the challenges of gathering data at this scale for robust snow leopard occupancy and related analysis and point to adapted methodological approaches.

Our findings support the proposition that prey presence is a key determinant of snow leopard site use [27,49]. This relationship was not observed at the camera trap level [47], highlighting that such relationships may only emerge at the larger scale. Blue sheep are widespread in QNNR, as evidenced by the detection of blue sheep signs in 76% of sampled grid cells. During winter, blue sheep are likely to be the main prey in the QNNR [50,51], given that other wild ungulate species, for example the larger white-lipped deer, are scarce in the area [52], and that other prey species, such as Himalayan marmots (*Marmota himalayana*), hibernate until early April. The strong link between snow leopard and blue sheep highlights the need for conservation programmes to include blue sheep, even though they are currently listed as a species of least concern under the IUCN red list and they are not identified as a protected species in China. In QNNR it remains unclear to what extent human activities pose a threat to blue sheep. For instance, in the absence of proper livestock management, increasing livestock density may reduce wild prey abundance, causing snow leopard to either engage in depredation of livestock or leave the area [49]. Livestock rearing is an important livelihood source for local people in QNNR [30] and livestock grazing was observed to be common throughout the reserve, albeit in less than 50% of grid cells. Other threats to prey include poaching, of which we observed some evidence, and disease [10,53]. We encountered one dead blue sheep with what appeared to be scabies (although this could not be confirmed by laboratory testing). There is a need for monitoring of blue sheep populations at the local level and for further research into the ecology of this critical prey species both in the study area and throughout the species’ range. Specifically, quantitative evidence is lacking on predator-prey densities and on the long-term population dynamics of the blue sheep [10].

Our models did not attribute a strong predictive power of the presence of livestock grazing or distance to mines (with high standard errors associated with these covariates). The pervasiveness of human activities across the landscape that we surveyed, the low number of sampled sites and the scale of our analysis [47] may have affected our ability to identify existing relationships. Seasonal considerations also apply. At the time of field work, herders may have been concentrating grazing activity within winter pastures located closer to their settlements, as reported in a survey we have previously undertaken in the area [30]. Use of pastures in the more remote and higher elevation sites was reported to primarily take place in the summer. This would result in greater spatial influence of small-stock grazing at that time of year. We did nonetheless observe a wide range of human disturbances associated with livestock grazing throughout the study area, including the presence of humans, dogs and fences. Herders commonly kept domestic dogs or maintained feral dogs near their homes or livestock. In addition wire fencing (c. 1 metre high, fixed with wooden poles) has been installed to define herding areas throughout QNNR. Encouragingly however a governmental ban on livestock grazing has been in place across certain sections of QNNR since 2012 (and is expected to be on-going until at least 2020), resulting in a reported decline in herding activities [30]. The consequences for snow leopards of these multiple forms of human activity and related livestock grazing policies merit, in our view, more detailed investigation.

Similarly mining was observed to occur throughout the nature reserve. We noted that it involved the development of significant transport infrastructure, including the building of new roads, leading to substantial movement of people and vehicles. Nonetheless, on occasion, we detected the presence of snow leopards less than one km from mines. It is possible that snow leopards can adapt to localized and concentrated human disturbances or move through affected areas, as observed with pumas and leopards [4,7], and occasionally even with dispersing tigers [54]. However, elevation complexity within the mountain range may reduce any impact of extractive developments. In addition, it should be recognized that we only measured snow leopard use of these sites, not their abundance, so our results do not necessary suggest that snow leopards can thrive in these disturbed environments. Finally we also acknowledge our inability to differentiate between the different types, scales and seasonal fluctuations of activities related to mining and their differential impact (e.g., through noise, pollution, movement) on habitat use. For example we were unable to capture the effect of non-active mines. These mines may be associated with some residual effects, even though we observed snow leopards tracks in abandoned mining sites. Small holder-mining activity would be expected to be greater during the summer, and may involve sites further from towns or settlements.

Attention is needed to avert any risks to snow leopards associated with expanding development activities, such as mining and road building. The economic incentives surrounding mineral extraction are considerable for provincial and national governments, and political sensitivity is necessary to effect any long-term changes. At the local level, however, simple measures could nonetheless be taken to alleviate any untoward environmental impacts. For instance in 2015 the QNNR authorities have reported encouraging measures to limit large-scale mining activities within the totality of the reserve and to expand its core area, where all human activities are prohibited. Additional measures targeted towards illegal small-scale mining activities are also needed, through better controls and the imposition of penalties for breaching existing regulations.

Mining is likely to be a growing concern in the snow leopard range. The implications are significant, especially in China, one of the world’s largest producers of raw materials [55]. Further investment is required to examine more closely trends in mining activity and potential channels of impact on snow leopard populations. This is an area where GPS collaring investigations may be helpful in tracking individual snow leopards movements and responses. Before and after assessments of new mining activities would also be valuable.

Non-invasive survey techniques combined with large-scale occupancy methods for felids pioneered with the tiger are generating important new insights in carnivore ecology and methods [38,39]. These methods need to be adapted to the snow leopard to account for the different life history characteristics and habitat between tigers and snow leopards. Our findings reaffirm the value of explicitly taking into imperfect detection and spatial-autocorrelation [29,38]. They also highlight some important issues that need to be addressed in this line of research. Previous snow leopard studies that have not used such methods may have substantially underestimated their range. By focusing on optimum snow leopard habitats, they may have missed key low-use areas that might act as conduits for dispersal and movement [18].

Firstly, the harsh and remote terrain of the snow leopard habitat severely limits survey size and reach, and increases costs. Due to limited site accessibility, we were unable to survey some more remote areas within Qifeng and our findings cannot be assumed to generalize to these areas without further verification. Alternative strategies would be required to survey the entire landscape, for example implementing a continuous transect segment over a 3-month survey period [56].

Secondly, efforts should be made to reduce all potential sources of bias related to data collection. Any errors in species identification should be accounted for by using field and analytical methods that can estimate species misidentification error, and correct for habitat occupancy estimates over larger landscape[57]. This can be achieved by cross-validating field judgements of fecal DNA samples in conjunction with laboratory techniques, and extrapolating on samples that fail to amplify. Similarly, intensive camera trapping in select number of sites can confirm true presence of an animal when tracks are thought to be present.

Thirdly, our simulation results highlight that future surveys of snow leopard site use should seek to increase the number of sampled sites in order to improve the precision and accuracy of occupancy and detection estimates. Even when detection rates are high, in the order of 0.68, large sample sizes are required. Our results suggest that a survey effort greater or equal to 80 sites would be required to obtain reasonably precise and accurate parameter estimates. A larger sample size should also allow for the application of two species co-occurrence models, which account for imperfect detection of both the species under study and other species, in our case prey or livestock. This would also allow for the inclusion of additional covariates, such as terrain heterogeneity and distance to roads, which would strengthen the analysis.

Our study marks a step forward in applying occupancy modelling to snow leopards at a larger scale and in assessing the influence of key determinants, including those related to human disturbances. These principles are applicable to wider “true occupancy” studies that should be conducted across the Reserve to identify key source populations of snow leopards and help determine areas for strict protection. Such core areas should be planned within a landscape framework, allowing for movement and dispersal of both snow leopard and prey [18].

## Conclusion

Improved knowledge about the effect of various forms and rapidly evolving nature of human encroachment in the snow leopard range is urgently needed for conservation planning at the local and global levels. Further studies should investigate how snow leopards are responding to shifting patterns of livestock rearing and to expanding economic development projects. Such studies need to be carefully designed and properly resourced to adequately address a number of critical methodological issues, many of which are specific to snow leopard research.

## Supporting Information

S1 File Estimation of the standard error of the mean snow leopard occupancy. (DOCX)

S2 File Simulation results to evaluate sample size adequacy. (DOCX)

S3 File Model-averaged estimates of site use and detection, including their respective standard errors, for snow leopards in Qilianshan National Nature Reserve, 2014. (DOCX)
