# Supplementary material for: Patterns of Snow Leopard Site Use in an Increasingly Human-Dominated Landscape
Source: PLoS One. 2016 May 12;11(5):e0155309. doi: 10.1371/journal.pone.0155309 (PMC4865053; doi:10.1371/journal.pone.0155309)
Supplement: S1 File — (DOCX) [file pone.0155309.s001.docx]

**S1 File: Estimation of the standard error of the mean snow leopard occupancy.**

The standard error estimate of the mean occupancy rate ($\hat{\bar{\psi}}$) and detection (${\hat{\bar{p}}}_{t(i)}$) was calculated using parametric bootstrapping following the methods of Karanth et al. (2011), where the number of sites was equal to 49.

$$\hat{SE}\left( \hat{\bar{\psi}} \right)=\frac{1}{49}\sqrt{\sum_{i=1}^{49} \hat{Var}\left( \hat{\psi}_{i} \right)+\sum_{i=1}^{49} \sum_{\begin{aligned} j=1 \\ j\neq i \end{aligned}}^{49} \hat{Cov}\left( \hat{\psi}_{i},\hat{\psi}_{j} \right)}$$

More specifically, parametric bootstrapping was used to estimate the covariance between the occupancy rates for any pair of sites (*i,j*), using the untransformed $\beta$ parameter estimates (e.g. $\hat{Cov}\left( \hat{\psi}_{i},\hat{\psi}_{j} \right)$ ) [1,2], derived from our Variance-Covariance matrix produced by the program PRESENCE [3] version 8.8. We simulated 1000 random deviates from a multivariate normal distribution using the MSBVAR package in R [4]. Site-specific probabilities of occupancy, *i*, were then computed utilizing the logit link function as follows:

$$\hat{\psi}_{i}= \frac{e^{x_{i}^{T_{\hat{\beta}}}}}{{{1+e}^{x_{i}^{T_{\hat{\beta}}}}}}$$

In the equation above $x^{T}$ is the vector of covariates used to determine snow leopard occupancy. Then for each estimated pair $\left( \hat{\psi}_{i},\hat{\psi}_{j} \right)$, where $i \neq j$, the covariance was computed as per [1]:

$$\hat{Cov}\left( \hat{\psi}_{i},\hat{\psi}_{j} \right) =\frac{\sum_{k=1}^{1000} \left( \hat{\psi}_{ik}- {\hat{\bar{\psi}}}_{i} \right)\left( \hat{\psi}_{jk}- {\hat{\bar{\psi}}}_{j} \right)}{999}$$

In the equation above, *k* indexes each vector of simulated $\beta$ random deviates.

The above procedure is relevant to any one particular model defined by a set of $\beta$ parameters. This procedure was repeated for each model. The same procedure was used for calculating the standard error estimate of the mean detection (${\hat{\bar{p}}}_{t(i)}$) rate.

**References**

1. Karanth KU, Gopalaswamy AM, Kumar NS, Vaidyanathan S, Nichols JD, et al. (2011) Monitoring carnivore populations at the landscape scale: occupancy modelling of tigers from sign surveys. J Appl Ecol 48: 1048–1056.

2. Efron B, R. Tibshirani (1986) Bootstrap methods for standard errors, confidence intervals, and other measures of statistical accuracy. Stat Sci 1: 54–77.

3. Hines JE (2006) PRESENCE2 - Software to estimate patch occupancy and related parameters. Available: http://www.mbr-pwrc.gov/software/presence.html.

4. R Core Team (2014) R: A Language and Environment for Statistical Computing. Available: http://www.r-project.org.
