# Supplementary material for: Patterns of Snow Leopard Site Use in an Increasingly Human-Dominated Landscape
Source: PLoS One. 2016 May 12;11(5):e0155309. doi: 10.1371/journal.pone.0155309 (PMC4865053; doi:10.1371/journal.pone.0155309)
Supplement: S2 File — (DOCX) [file pone.0155309.s002.docx]

**S2 File: Simulation results to evaluate sample size adequacy**

We carried out a simulation study to investigate the effect of sample size (number of sites/grid cells) on the bias and precision of $\hat{\bar{\psi}}$ estimates [1]. We simulated data in the program GENPRES [1] using the “single-season-spatial-correlation” model [2]. We used our final model estimates of $\hat{\bar{\psi}}$ , ${\hat{\bar{p}}}_{t}$,$\theta, \theta^{'} \mathrm{and} \theta_{0}$as the true values to simulate data and varied the number of survey sites (20, 30, 40, 50, 60, 70, 80, 90, 100, 200 sites). In all simulations $\theta, \theta^{'} \mathrm{and}\theta_{0}$were maintained constant at 0.19, 0.87, and 0.18 respectively. Site-level occupancy was set at 0.75; detection probability at 0.68; and the number of spatial replicates at 14.

One thousand simulations replicates (n=1000) were conducted and the resulting estimates of $\hat{\psi}$ were noted. For each simulation we approximated bias and precision by calculating the root mean square error:

$\hat{\mathrm{RMSE}}=$ $\sqrt{\hat{Var}\left( \hat{\psi} \right)+ \left( {\hat{\bar{\psi}}}_{true}-\hat{\psi} \right)^{2}}$

Low RMSE indicates higher quality of the estimate (a trade-off between variance and bias). Our simulation results indicate that our model estimates of occupancy were not of optimal quality (RMSEs for occupancy equal to 0.07 for 50 grid cells) and that RMSE decreased with the increase in the number of sampling sites (Table 1 & 2). The largest decrease in RMSE for all parameters occurred when the number of sites increased from 20 to 30.

**Table 1: The relationship between the number of sampled sites and root mean square error (RMSE) of occupancy, detection estimates generated from 1000 simulations for the single-season-spatial-correlation model.**

| No.  Sites | $\hat{\psi}$ | $\hat{SE}\left( \hat{\psi} \right)$ | RMSE $(\hat{\psi})$ | $\hat{p}_{t}$ | $\hat{SE}\left( \hat{p}_{t} \right)$ | $\mathrm{RMSE}\left( \hat{p}_{t} \right)$ |
| --- | --- | --- | --- | --- | --- | --- |
|  |  |  |  |  |  |  |
| 20 | 0.7722 | 0.1196 | 0.0926 | 0.7174 | 0.1062 | 0.0850 |
| 30 | 0.7576 | 0.0922 | 0.0926 | 0.7125 | 0.0786 | 0.0850 |
| 40 | 0.7494 | 0.0799 | 0.0799 | 0.7115 | 0.0653 | 0.0725 |
| 50 | 0.7511 | 0.0701 | 0.0701 | 0.7093 | 0.0604 | 0.0671 |
| 60 | 0.7475 | 0.0663 | 0.0663 | 0.7086 | 0.0540 | 0.0611 |
| 70 | 0.7445 | 0.0620 | 0.0622 | 0.7062 | 0.0504 | 0.0569 |
| 80 | 0.7451 | 0.0537 | 0.0539 | 0.7063 | 0.0470 | 0.0538 |
| 90 | 0.7432 | 0.0532 | 0.0536 | 0.7057 | 0.0448 | 0.0516 |
| 100 | 0.7423 | 0.0527 | 0.0532 | 0.7095 | 0.0405 | 0.0501 |
| 200 | 0.7388 | 0.0351 | 0.0369 | 0.7044 | 0.0282 | 0.0373 |
|  |  |  |  |  |  |  |

**Table 2: The relationship between the number of sampled sites and root mean square error (RMSE) of the segment-level occupancy parameter estimates generated from 1000 simulations for the single-season-spatial-correlation model.**

| No.  Sites | $\hat{\theta}$ | $\hat{SE}\left( \hat{\theta} \right)$ | RMSE $(\hat{\theta})$ | $\hat{\theta}$’ | $\hat{SE}\left( \hat{\theta}’ \right)$ | $\mathrm{RMSE}\left( \hat{\theta}’ \right)$ | $\hat{\theta}_{0}$ | $\hat{SE}\left( \hat{\theta}_{0} \right)$ | RMSE ($\hat{\theta}_{0}$) |
| --- | --- | --- | --- | --- | --- | --- | --- | --- | --- |
|  |  |  |  |  |  |  |  |  |  |
| 20 | 0.2197 | 0.0647 | 0.0602 | 0.8345 | 0.0891 | 0.0733 | 0.2197 | 0.0647 | 0.0659 |
| 30 | 0.2215 | 0.0512 | 0.0602 | 0.8398 | 0.0668 | 0.0733 | 0.2215 | 0.0512 | 0.0659 |
| 40 | 0.2221 | 0.0463 | 0.0564 | 0.8434 | 0.0564 | 0.0623 | 0.2221 | 0.0463 | 0.0626 |
| 50 | 0.2222 | 0.0404 | 0.0517 | 0.8421 | 0.0508 | 0.0580 | 0.2222 | 0.0404 | 0.0585 |
| 60 | 0.2203 | 0.0377 | 0.0484 | 0.8444 | 0.0450 | 0.0518 | 0.2203 | 0.0377 | 0.0552 |
| 70 | 0.2196 | 0.0348 | 0.0458 | 0.8456 | 0.0430 | 0.0494 | 0.2198 | 0.0348 | 0.0529 |
| 80 | 0.2215 | 0.0346 | 0.0468 | 0.8463 | 0.0409 | 0.0473 | 0.2215 | 0.0346 | 0.0540 |
| 90 | 0.2208 | 0.0319 | 0.0444 | 0.8466 | 0.0385 | 0.0451 | 0.2208 | 0.0319 | 0.0518 |
| 100 | 0.2209 | 0.0282 | 0.0418 | 0.8448 | 0.0350 | 0.0432 | 0.2209 | 0.0282 | 0.0497 |
| 200 | 0.2202 | 0.0206 | 0.0366 | 0.8482 | 0.0238 | 0.0322 | 0.2203 | 0.0206 | 0.0452 |
|  |  |  |  |  |  |  |  |  |  |

**References**

1. Bailey LL, Hines JE, Nichols JD, MacKenzie DI (2007) Sampling design trade-offs in occupancy studies with imperfect detection: examples and software. Ecol Appl 17: 281–290.

2. Hines JE, Nichols JD, Royle JA, MacKenzie DI, Gopalaswamy AM, et al. (2010) Tigers on trails: occupancy modeling for cluster sampling. Ecol Appl 20: 1456–1466.
