# Supplementary material for: Patterns of Snow Leopard Site Use in an Increasingly Human-Dominated Landscape
Source: PLoS One. 2016 May 12;11(5):e0155309. doi: 10.1371/journal.pone.0155309 (PMC4865053; doi:10.1371/journal.pone.0155309)
Supplement: S3 File — (DOCX) [file pone.0155309.s003.docx]

**S3 File: Model-averaged estimates of site use and detection, including their respective standard errors, for snow leopards in Qilianshan National Nature Reserve, 2014.**

|  | Model ^*^ | ${\hat{\bar{\psi}}}_{i}$ | $\hat{SE}\left( {\hat{\bar{\psi}}}_{i} \right)$ | ${\hat{\bar{p}}}_{t(i)}$ | $\hat{SE}\left( {\hat{\bar{p}}}_{t(i)} \right)$ |  |  |  |
| --- | --- | --- | --- | --- | --- | --- | --- | --- |
|  |  |  |  |  |  |  |  |  |
| 1 | ψ(BS),θ’(.),θ(.),θ_0_(.),p_t_(M) | 0.7485 | 0.0912 | 0.6832 | 0.0842 |  |  |  |
| 2 | ψ(BS+L),θ’(.),θ(.),θ_0_(.),p_t_(M) | 0.7534 | 0.1149 | 0.6823 | 0.0868 |  |  |  |
| 3 | ψ(BS+M),θ’(.),θ(.),θ_0_(.),p_t_(M) | 0.7528 | 0.0971 | 0.6826 | 0.0824 |  |  |  |
| 4 | ψ(global),θ’(.),θ(.),θ_0_(.),p_t_(M) | 0.7554 | 0.1163 | 0.6819 | 0.0817 |  |  |  |
|  | *Model Averaged* | 0.7497 | 0.1010 | 0.6814 | 0.0843 |  |  |  |

^*^Covariates considered Mine (M), Blue Sheep (BS) and Livestock (L).

ψ: the probability of snow leopard site use

θ’: Probability a snow leopard use of a transect segment conditional on snow leopards did use the previous segment

θ: Probability a snow leopard use of a transect segment conditional on snow leopards did not use the previous segment

θ_0_: Probability a snow leopard use of the first transect segment conditional on the segment before the first segment is occupied

p_t_: Probability of detecting a snow leopard in a transect segment conditional on snow leopards used the transect segment

Where ${\hat{\bar{\psi}}}_{i}$ was defined as the estimated snow leopard occupancy rate for a cell *i*.

Where ${\hat{\bar{p}}}_{t(i)}$ is defined as the estimated snow leopard detection rate for replicate *t* and cell *i*.

Model-averaged estimates with unconditional standard errors.
